# Accessible online neuroplasticity-targeted training for children with ADHD

**DOI:** 10.1186/1753-2000-7-38

**Published:** 2013-11-14

**Authors:** Jyoti Mishra, Michael M Merzenich, Rajesh Sagar

**Affiliations:** 1 Department of Neurology, Physiology and Psychiatry, Sandler Neurosciences Center, University of California, San Francisco, CA 94158, USA; 2 Brain Plasticity Institute, PositScience, San Francisco, CA 94108, USA; 3 Department of Psychiatry, All India Institute of Medical Sciences, New Delhi 110029, India

**Keywords:** Attention Deficit Hyperactivity Disorder, ADHD, Cognitive training, Neuroplasticity, Neurotherapeutic, Global mental health

## Abstract

Childhood Attention Deficit Hyperactivity Disorder (ADHD) is a growing mental health concern worldwide. Effective, accessible and low-cost therapeutics for the disorder are urgently needed. Here we introduce a novel internet-based cognitive training intervention: Online Neuroplasticity-based Training for the Remediation of ADHD in Children (ONTRAC). The intervention is deployed in the home setting; it is customized to the cognitive capacities of each child and progressively improves performance in the specific neuro-cognitive domains deficient in ADHD. A feasibility trial of ONTRAC is being conducted in a resource limited clinical setting in New Delhi, India and is an exemplar of hi-tech global psychiatry.

## Correspondence

Nearly 10% of children worldwide are being diagnosed with ADHD [1-3]. With the new DSM-V diagnostic criteria [4] adopted around the world, childhood ADHD rates are further likely to increase, and the disorder is poised to become a global mental health epidemic. Given the immense socio-economic and healthcare burden posed by the disorder, developing effective and accessible treatments is an immediate need.

Stimulant medications remain the conventional first line of treatment in many countries, partly due to unavailability of any other reasonable treatment option [5-7]. However, research shows that while these medications improve on-task attention and provide respite in the short-term, significant behavioral impairments and poor academic performance persist over the long-term and medication side effects accumulate [8]. Further, in resource-limited global settings, there is poor access to affordable medications, mental health specialists and long-term care, and social stigma surrounds diagnosis and pharmacological treatment of mental disorders in many countries [9,10]. In general, parents worldwide prefer treatments for their children that are accessible, affordable, sustainable within the family’s lifestyle and those found to be effective in clinical trials. In this context, cognitive training (CT) is emerging as a promising new alternative and/or medication-adjunct treatment option for ADHD [11,12].

The advantage of modern CT is that it is internet-based and hence accessible from any remote setting. Scientifically, we can now develop CT that is specifically targeted to the core neuropsychological and cognitive impairments observed in a neuropsychiatric disorder or clinical population [13-17]. Using principles that drive neuroplasticity [18], we have recently developed such a novel CT aimed at improving cognition in children with ADHD: Online Neuroplasticity-based Training for the Remediation of ADHD in Children (ONTRAC). ONTRAC incorporates technological advances that facilitate integration within clinical care: it is internet-deployed on secure servers so that children can conveniently access the training from any computer in their home setting; it automatically tracks compliance and performance progress in training session data uploads to the cloud, and it is scalable and sustainable as a single clinician can remotely monitor the training progress of hundreds of children simultaneously.

Scientifically, ONTRAC features cognitive personalization as the training challenges are adaptively modified in real-time based on the individual’s current performance capabilities. Most importantly, ONTRAC is custom-designed to selectively target ADHD-specific deficits in five critical neuro-cognitive domains of alertness, sustained and selective attention, working memory, impulsive response control and suppression of distracting interference. No prior ADHD CT program has comprehensively targeted all of these cognitive domains; especially, the key deficits observed in suppression of distracting information have not been targeted.

ONTRAC is being currently evaluated in a recently initiated randomized controlled feasibility-efficacy trial in New Delhi, India [19]. All study procedures and written informed consents obtained from participating families were approved by the Institution Ethics Committee at the All India Institute of Medical Sciences, New Delhi (Approval # IEC/NP-359/2011 & RP-03/2012). This global setting, with limited specialized psychiatric care yet widespread internet-connectivity, is well suited for assessing the utility of ONTRAC to impact ADHD when access to clinical resources is sparse [20]. Adolescent children access and progress through training from their home setting performing 30 hours of CT over 6 months. An age and medication-status matched active control group engages in 30 hours of non-therapeutic video game play over the same time period. Training parameters such as content, duration and frequency have all been carefully titrated to maximize engagement, compliance and efficacy. Changes in ADHD symptoms and in underlying neuropsychological function are being measured in 3-monthly visits: pre-, mid-, post-training and in a 6-month follow-up.

Results pending, the ONTRAC trial illustrates in principle how modern technology and clinical neuroscience can be integrated for the development and evaluation of a 21st century personalized neurotherapeutic that may benefit many individuals with neuropsychiatric impairments in remote and resource-limited settings. We are aware that we may not achieve positive long-term real-life impacts in a first trial. Yet, the technology base of ONTRAC allows rapid iterative software development cycles. When rapid technology development is coupled with systematic and well-controlled clinical trial evaluations, it is envisaged that research efforts would ultimately converge onto an efficacious cognitive training program in the near future that has global positive and sustaining impact on ADHD in children.

## Abbreviations

ADHD: Attention Deficit Hyperactivity Disorder; CT: Cognitive training; ONTRAC: Online Neuroplasticity-based Training for the Remediation of ADHD in Children.

## Competing interests

JM is a part-time scientist at the Brain Plasticity Institute, PositScience and MM is President and Founder of Brain Plasticity Institute, PositScience, a company that develops cognitive training software.

## Authors’ contributions

All authors contributed to the writing and approval of this letter.
